# Genetic Diversity and Azole Fungicide Sensitivity in *Pseudocercospora musae* Field Populations in Brazil

**DOI:** 10.3389/fmicb.2020.00099

**Published:** 2020-02-04

**Authors:** Fabiane S. D. Brito, Jansen R. P. Santos, Vânia C. R. Azevedo, Yslai S. Peixouto, Saulo A. de Oliveira, Cláudia F. Ferreira, Fernando Haddad, Edson P. Amorim, Bart Fraaije, Robert N. G. Miller

**Affiliations:** ^1^Instituto Federal Goiano, Posse, Brazil; ^2^Department of Phytopathology, University of Brasília, Brasília, Brazil; ^3^Department of Plant Genetics, Embrapa Recursos Genéticos e Biotecnologia, Brasília, Brazil; ^4^Department of Plant Breeding and Phytopathology, Embrapa Mandioca e Fruticultura, Cruz das Almas, Brazil; ^5^Department of Biointeractions and Crop Protection, Rothamsted Research, Harpenden, United Kingdom

**Keywords:** *Pseudocercospora musae*, *Musa* spp., Sigatoka leaf spot, population genetics, SSR markers, demethylation inhibitor fungicide resistance

## Abstract

*Pseudocercospora musae*, causal agent of Sigatoka leaf spot, or yellow Sigatoka disease, is considered a major pathogen of banana (*Musa* spp.). Widely disseminated in Brazil, this study explored the genetic diversity in field populations of the pathogen from production areas in the Distrito Federal and the States of Bahia, Minas Gerais, and Rio Grande do Norte. Resistance to demethylation inhibitor (DMI) fungicides was also examined. For 162 isolates from 10 banana growing regions, analysis of mating type idiomorph frequency was conducted, together with estimation of genetic diversity at 15 microsatellite loci. A total of 149 haplotypes were identified across the examined populations, with an average genetic diversity of 4.06. In general, populations displayed 1:1 proportions of idiomorphs MAT1-1 and MAT1-2, providing evidence for sexual recombination. Multilocus linkage disequilibrium also indicated asexual reproduction contributing to the genetic structure of certain populations. AMOVA revealed that 86.3% of the genetic differentiation of the pathogen occurred among isolates within populations. Discriminant Analysis of Principal Components (DAPC) identified six most probable genetic groups, with no population structure associated with geographic origin or collection site. Although genetic similarity was observed among certain populations from different states, data revealed increasing genetic differentiation with increasing geographic distance, as validated by Mantel’s test (*r* = 0.19, *P* < 0.001). On the basis of DMI fungicide sensitivity testing and *CYP51* gene sequence polymorphism, isolates from the Distrito Federal separated into two main groups, one with generally higher EC_50_ values against eight DMI fungicides. A clear phenotype-to-genotype relationship was observed for isolates carrying the CYP51 alteration Y461N. Conventionally adopted fungicides for control of Sigatoka leaf spot are likely to be overcome by combined sexual and asexual reproduction mechanisms in *P. musae* driving genetic variability. Continued analysis of pathogen genetic diversity and monitoring of DMI sensitivity profiles of Brazilian field populations is essential for the development of integrated control strategies based on host resistance breeding and rational design of fungicide regimes.

## Introduction

*Pseudocercospora musae* (Zimm.) Deighton [sexual morph: *Mycosphaerella musicola* R. Leach ex J. L. Mulder], causal agent of Sigatoka leaf spot, or yellow Sigatoka disease, is one of the main pathogens affecting banana fruit production worldwide (Arzanlou et al., 2007). Initially reported in Indonesia in 1902, the fungus spread to most banana production areas by the 1960s (Jones, 2009). The main symptoms of this disease are necrotic leaf spots, which lead to reduced photosynthetic capacity and a subsequent reduction in fruit size and number per bunch (Hayden et al., 2005; Arzanlou et al., 2007).

Sigatoka leaf spot was first reported in Brazil in 1944 in the Amazon region (Kimati and Galli, 1980; Pereira et al., 1998), with subsequent spread to all states. Although control of the disease relies upon either planting of resistant materials or frequent fungicide application, appropriate commercial varieties resistant to the disease are lacking and producers note a reduction in control efficiency of fungicides. Agrochemical control is currently dependent upon DMI fungicides (imidazoles and triazoles) (Cañas-Gutiérrez et al., 2009; Churchill, 2011). Although resistance to such azoles has been reported for numerous plant pathogens (reviewed by Cools et al., 2013), the available records for *P. musae* populations relate only to propiconazole (Peterson et al., 2003). Azole fungicides act through the inhibition of sterol 14α-demethylase (CYP51), a key enzyme in ergosterol biosynthesis, regulating membrane fluidity and function and essential for cell survival (Lepesheva and Waterman, 2004). Reduced sensitivity of fungal pathogens to azoles can be related to mutations and over-expression of the *CYP51* gene. In *Pseudocercospora fijiensis*, for example, the causal agent of black leaf streak or black Sigatoka disease in *Musa*, which is phylogenetically closely related to *P. musae*, resistance is associated with single or multiple mutations underlying at least seven CYP51 amino acid substitutions (Y136F, A313G, H380N, A381G, Y461D, G462A, Y463D/H/N/S) which likely affect azole binding. Resistance in this species is also associated with a 19 bp repeat elements in the CYP51 promoter region linked to gene over-expression (Cañas-Gutiérrez et al., 2009; Chong Aguirre, 2016). In contrast to *P. fijiensis*, limited monitoring of fungicide sensitivity has been conducted in *P. musae*, with potential mutations associated with resistance to azoles yet to be characterized.

Sexual recombination in fungi is a process that can occur once or several times during a crop growing season, as well as during off-season periods in the absence of primary hosts (Meng et al., 2015). Along with factors such as genetic drift, mutation and parasexuality (Brent and Hollomon, 2007), sexual recombination also contributes toward genetic variability in heterothallic fungi. For this, plasmogamy, meiosis and exchange of genetic material can only occur between compatible mating-type isolates present at a particular location. Following the detection of mating pheromones from an opposite mating-type isolate, cellular responses will lead to mating between the compatible isolates (Coppin et al., 1997; Kronstad and Staben, 1997). The mating type locus in heterothallic filamentous ascomycetes, such as members of the genus *Pseudocercospora*, contains one of two dissimilar, non-allelic gene sequences, referred to as idiomorphs, which occupy a common chromosomal position in the genome for all isolates of a given species (Conde-Ferráez et al., 2007; Arzanlou et al., 2010). Mating type idiomorphs in complementary heterothallic isolates of a particular species are known as MAT1-1 and MAT1-2. Isolates of the former type have a single gene (*MAT1-1-1*), which encodes a protein containing an alpha domain. The latter isolates possess a *MAT1-2-1* gene, encoding a protein with a high-mobility group domain (HMG). Both genes encode transcription factors which are known to regulate the sexual cycle through control of signal transduction involved in mating identity (Wirsel et al., 1998; Nolting and Pöggeler, 2005; Conde-Ferráez et al., 2007). Mating type idiomorph-harboring genome regions appear to be conserved among closely related species in the genus (Arzanlou et al., 2010; Conde-Ferráez et al., 2010; Kim et al., 2013). For *P. fijiensis* (Zhan et al., 2002; Conde-Ferráez et al., 2010; Queiroz et al., 2013) and *Zymoseptoria tritici* (Gurung et al., 2011), analysis of the mating type region has often revealed a frequency close to 1:1 of *MAT1-1* and *MAT1-2*, indicating regular cycles of sexual recombination during the crop growing season. Unlike these Dothideomycete fungal species, analysis of the distribution of mating type idiomorphs in *P. musae* populations has so far been limited (Gomes et al., 2017).

Molecular-based approaches have been employed for analysis of the population genetic structure of different *Pseudocercospora* species at local, regional and continental scales. Informative markers have been based initially on random amplified polymorphic DNA (RAPD) (Moreira et al., 2003), amplified fragment length polymorphism (AFLP), restriction fragment length polymorphism (RFLP) (Carlier et al., 1996; Hayden et al., 2005), ERIC-PCR (Silva et al., 2014), inter-simple sequence repeats (ISSR) (Peixouto et al., 2015) and microsatellites or simple sequence repeats (SSRs) (Gurung et al., 2011). Whilst the latter are co-dominant markers appropriate for population genetic studies (Selkoe and Toonen, 2006), application in analysis of genetic diversity of *P. musae* populations has been limited (Molina et al., 2001; Moreira et al., 2003; Zapater et al., 2008; Gomes et al., 2017).

This study was conducted to determine the genetic diversity and structure in populations of *P. musae* from important banana production areas in Brazil across the Distrito Federal and the States of Bahia, Minas Gerais, and Rio Grande do Norte, ranging in distance from 22 to 1900 km. Analyses of dispersal and genetic recombination processes were based on mating type idiomorph frequencies and SSR-based genetic diversity within and among populations. Additional investigation of sensitivity to DMI fungicides and analysis of potential mutations in the *CYP51* gene was conducted on isolates from sampled populations.

## Materials and Methods

### Isolate Sampling

**FIGURE 1:**
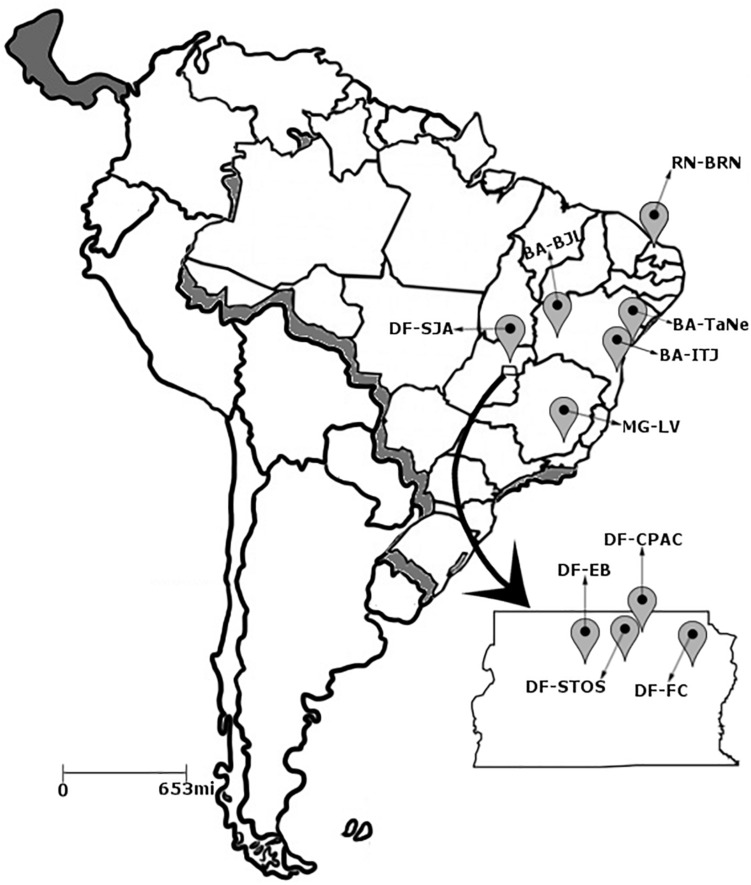
Distribution of the 10 collection sites of the *Pseudocercospora musae* isolates. Distrito Federal: (1) EB – Estação Biológica; (2) CPAC – Embrapa Cerrados; (3) FC – Fazenda Canaã; (4) SJA – São João da Aliança; (5) STO – Chácara Santos. Bahia; (6) BJL – Bom Jesus da Lapa; (7) TaNe – Tancredo Neves; (8) ITJ – Itajuípe. Minas Gerais; (9) LV – Lavras. Rio Grande do Norte; (10) BRN – Baraúna.

*Pseudocercospora musae* isolates were collected from infected banana leaf material with typical Sigatoka leaf spot symptoms (Brazilian National System for the Management of Genetic Heritage SISGEN registration number A2B3786). Material originated from 10 different zones across Brazil, distributed in the Distrito Federal and surroundings (DF), as well as the states of Bahia (BA), Minas Gerais (MG) and Rio Grande do Norte (RN) (Figure 1). Isolates were collected from a range of different susceptible *Musa* genotypes across the sampled production areas (Supplementary Table S1). At each location, within an area of approximately 50 m^2^, a total of five banana plants were selected for sampling, representative of each border and the central region. Two leaves displaying disease symptoms were selected per plant and 20 cm^2^ fragments from the top and bottom of each leaf selected. Collected material was surface sterilized using sequential washes of tap water, ethanol (75%) and distilled water. Leaf lesions were incubated in Petri dish moist chambers for 48 h then observed under stereoscope microscopy to check for sporodochia. In order to obtain monosporic isolates, conidia were transferred aseptically from sporodochia to slides containing water-agar blocks (1 cm^2^) and subsequently to solid V8 agar medium (9 g of agar, 1 g of CaCO_3_, 50 mL of V8 juice, 450 mL of distilled water, and 0.167 g chloramphenicol). All pure cultures were preserved in sterile distilled water (Castellani, 1939) and on sterile filter paper.

### DNA Extraction and Molecular Identification

Genomic DNA was extracted from mycelia for each isolate following a standard protocol (Doyle and Doyle, 1990). Isolates were identified to species level based on sequence data for the ribosomal DNA Internal Transcribed Spacer, comprising ITS1, 5.8S and ITS2 regions (ITS rDNA) (White et al., 1990), together with a portion of the actin gene (ACT) (Carbone and Kohn, 1999) and the histone H3 gene (HIS) (Crous et al., 2004). PCR conditions, as well as subsequent BLASTn-based sequence alignment and identification, were carried out as described by Crous et al. (2004). The sequences for the rDNA ITS region, ACT, and HIS were aligned with corresponding sequences for isolates from different species within the Sigatoka disease complex (*P. musae*, *P. fijiensis*, and *Pseudocercospora eumusae*) using the “Muscle” algorithm in the software MEGA, v.7. Phylogenetic analyses were conducted based on Bayesian inference, with the evolution model for each single region chosen using hierarchical likelihood tests considering the Akaike Information Criterion (AIC) within the program MrModeltest, v.2.3. For each target region, consensus trees were generated using the program MrBayes, v.3.2, with 5,000,000 generations (convergence 0.001) through three independent runs; each using four Markov Monte Carlo Chains (MCMC), two hot and two cold chains, a sampling of a tree every 1,000 generations, and burn-in of the initial 1,250,000 generations. Concatenated data was used to conduct a multilocus phylogenetic analysis, based on the same criterion for the Bayesian inference described above. Trees were visualized and edited using the program FigTree, v.1.3.1. (Rambaut, 2010).

### Characterization of Mating Type Genes

In order to determine the distribution of mating type idiomorphs in each population, the *MAT1-1-1* and *MAT1-2-1* genes were amplified using primers designed by Conde-Ferráez et al. (2010). PCR reactions were performed in a 25 μl volume using 10 ng of genomic DNA, 1 X buffer, 3 mM MgCl_2_, 0.2 mM dNTPs, 0.8 M each primer and 0.35 U Platinum Taq DNA polymerase (Invitrogen). All thermocycling reactions were carried out using the amplification program: 94°C for 5 min, 39 cycles of 95°C for 1 min, primer annealing at 63°C for 40 s and 72°C for 40 s, followed by a final extension of 7 min at 72°C. To confirm specific amplification of mating type idiomorphs, representative amplicons were purified with ExoSAP (Invitrogen, Carlsbad, CA, United States), sequenced on an ABI 3730 sequencer (Applied Biosystems, Foster City, CA, United States), and analyzed via BLASTn for comparison with the sequence of alpha and HMG conserved domains of *P. musae* available at NCBI^1^.

### Identification of Haplotypes

The genetic structure of *P. musae* populations (POPs) was analyzed using 15 SSR loci (Supplementary Table S1) specific to *P. musae* (Molina et al., 2001; Zapater et al., 2008). Each PCR reaction (13 μL) contained 15 ng genomic DNA; 1X buffer (10 mM Tris–HCl, pH 8.3; 50 mM KCl); 0.25 mM dNTPs; 0.2 μM each primer; 0.25 mg/ml BSA and 1 U Platinum Taq DNA polymerase (Invitrogen, Waltham, MA, United States). Thermocycling amplifications were carried out with a program of 5 min at 94°C for initial denaturation, followed by 34 cycles of 1 min at 95°C, an annealing step (50–60°C) of 1 min and 1 min and 30 s at 72°C, completed by a final 8 min extension at 72°C. To enable accurate genotyping based on the SSR alleles, PCR products were separated on an ABI 3730 sequencer with primers labeled with different fluorochromes (HEX and 6-FAM), in multiplex sets. Applied mixtures contained 18 μL Hidi, 1 μL of internal marker (ROX) and 1 μL of PCR product, denatured for 5 min at 95°C. Detection of fluorescence peaks and genotyping analysis were performed using the software GeneMapper, version 4.1 (Applied Biosystems, Foster City, CA, United States).

### Fungicide Sensitivity Testing

*In vitro* sensitivity testing of isolates was carried out in 96-well plate format according to Fraaije et al. (2012). Wells of flat-bottomed microtiter plates (655180, Greiner Bio-One, Frickenhausen, Germany) were filled with 100 μL aliquots of 2× Sabouraud Dextrose Liquid Medium (SDLM, Oxoid, Basingstoke, United Kingdom) un-amended and amended with 2.5-fold dilution series of fungicides (11 different concentrations). The final test concentration for epoxiconazole, pyrifenox, propiconazole, prochloraz and fluquinconazole ranged from 0.000105 to 1.0 mg L^–1^, for tebuconazole and cyproconazole from 0.001049 to 10 mg L^–1^, and for triadimenol a range of 0.005243–50 mg L^–1^. Prior to dilution in media, chemicals were dissolved in dimethyl sulphoxide (DMSO). Following 20 days growth on V8 medium, mycelial fragment suspensions were prepared by macerating two mycelial areas, of approximately 1 square cm diameter per isolate, in 3 mL of sterile distilled water using a mortar and pestle. As inoculum, 100 μL aliquots of mycelial suspensions (20-fold dilutions in water) were added to each well containing liquid medium. Plates were incubated for 7 days at 25°C and growth measured by absorbance readings at 630 nm using a FLUOstar OPTIMA microplate reader (BMG Labtech GmbH, Offenberg, Germany) in well-scanning mode with a 2 × 2 matrix of scanning points of 3-mm diameter. Fungicide sensitivities were determined as 50% effective concentration (EC_50_ in μg ml^–1^) using a dose-response relationship (4-parameter fit) determined with the OPTIMA Software.

### Sequence Analysis of the *CYP51* Gene

Potential genetic alterations in the *P. musae CYP51* gene target of the DMI fungicides were verified in complete and partial sequences of the gene, amplified using the primer pair combinations: MMFOR2 (CAA TGG GA/TC TCC TCC AGG A)/MMREV1 (TTG ACG CTG CCA/G TCC/G ACA TTG TA), MMFOR1 (GGA GGA GCG CTT TTG CTT CGG CTT)/MMREV5 (TAT GGA CAC GCC GAT AAA GAT GCT) and MMFOR8 (CGA CAC AGG TGC ATC GGT GAG CA)/MMREV8 (ATT TCT TTT CTC TCC TCT CCC ATC). Primers were designed based on *CYP51* gene sequences available from *Z. tritici* and *P. fijiensis* reference genomes^2^. PCR reactions were carried out on a Biometra T3000 thermocycler (Biotron, Göttingen, Germany) in a final volume of 50 μL containing 50 ng of fungal template DNA. For primer pair MMFOR2/MMREV1, PCRs contained 0.5 μM for each primer and 200 μM dNTP, 1× Phusion HF buffer, and 1.0 unit of Phusion High Fidelity DNA polymerase (New England Biolabs, Ipswich, MA, United States). Amplification conditions comprised 98°C for 30 s, followed by 40 cycles at 98°C for 10 s, 50°C for 20 s, and 72°C for 1 min with a final DNA extension at 72°C for 5 min. For primer pairs MMFOR1/MMREV5 and MMFOR8/MMREV8, PCR reactions contained 1.0 μM for each primer and 200 μM dNTP, 1× of Easy-A reaction buffer and 2.5 units of Easy-A High Fidelity PCR cloning enzyme (Agilent Technologies, Cedar Creek, United States). Amplifications were conducted at 95°C for 2 min, followed by 40 cycles at 95°C for 10 s, 60°C for 20 s, and 72°C for 2 min, with a final DNA extension at 72°C for 10 min. PCR products were sequenced by MWG Eurofins Genomics GmbH (Ebersberg, Germany). Sequences were assembled and aligned with Geneious v.6.1.4 software (Biomatters Ltd., Auckland, New Zealand), and amino acid substitutions determined after sequence analysis.

### Genetic Diversity Analyses

Following construction of an allele size matrix of multilocus SSR data, analysis of haplotypes and frequencies in each population, as well as AMOVA analyses, were all conducted using the R program with the POPPR and Vegan packages (Oksanen et al., 2013; Kamvar et al., 2014; R Development Core Team, 2014). Genetic differentiation between states was based on Jost’s (2008) D statistic (*D*_*EST*_) as an appropriate estimator for Wright’s *F*_*ST*_ or related *G*_*ST*_. Variance components and genetic differentiation values were tested by 1000 permutations for the haplotypes among the 10 populations, to estimate significance levels (*P* ≤ 0.05). The number of migrants per generation (*N*_*m*_) were calculated using the formula *N*_*m*_ = 0.5 (1 − *D*_*EST*_)/*D*_*EST*_. Genotypic diversity, richness and evenness were measured using the Shannon Wiener (H’) and Hill’s (N_1_) indexes. In order to determine the contribution of sexual reproduction or clonal spread in the populations, multilocus linkage-disequilibrium analyses were conducted across microsatellite loci using the index of association (*I*_*A*_) and a standardized version (*r*^–^*_*d*_*) that accounts for number of loci (Agapow and Burt, 2001), with *P* values obtained after 1000 permutations. Index values of zero are expected for the null hypothesis of linkage or gametic equilibrium under random mating, with statistically significant values above zero indicative of infrequent or an absence of sexual recombination. A minimum spanning network of the populations was also constructed to determine haplotype relationships, according to Bruvo’s distance (Bruvo et al., 2004). Cluster analysis to infer population genetic structure and the level of potential admixture among populations were also calculated using the R program, version 2.3.4 (Pritchard et al., 2000). Correlation between genetic and geographic distance among isolates was analyzed using the Mantel test (Mantel, 1967) using the package “ade4” within the R program (R Development Core Team, 2014). For this, a geographical distance matrix was generated by calculating the distance between isolates based on geographical coordinates, which was then compared with a matrix of genetic distances. In order to investigate the population structure of *P. musae*, *K*-means hierarchical clustering and Discriminant Analysis of Principal Components (DAPC) (Jombart et al., 2010) were performed. Frequencies of mating type idiomorphs for each *P. musae* population were calculated using the chi-square (χ2) test to determine significant deviation from the expected 1:1 ratio (Conde-Ferráez et al., 2010) using the SAS statistical package software (SAS Institute, 2012).

## Results

### Identification

A total of 162 *P. musae* isolates were obtained from different geographic regions in Brazil, from the Distrito Federal and the states of Bahia, Minas Gerais, and Rio Grande do Norte. Sample locations were separated from each other by distances ranging from 22 to 1900 km. Across the locations, *P. musae* isolates were obtained from *Musa* cultivars Maravilha, Fhia 17, Fhia 02, Cavendish Grande Naine, Thap Maeu, Prata, Prata comum, Pavocan, Maça, Prata anã, Prata comum, and Terra (Supplementary Table S1). All isolates displayed conidia typically cylindric to obclavate in shape, pale brown to olivaceous in color, aseptate, straight or curved, and with indistinct basal hila, in agreement with morphological descriptors for the species (Crous and Mourichon, 2002). Specific PCR products of the rDNA ITS region, with the expected size of 600 bp for *Cercospora*-like fungi (Crous et al., 2011), were amplified from all *P. musae* isolates. Similarly, specific amplification of the *ACT* and *HIS* gene fragments resulted in expected product sizes of 300 and 200 bp, respectively.

Representative sequences for the rDNA ITS region, ACT and HIS genes were deposited in GenBank with accession numbers KP996493, KP996494, and KP996495, respectively. BLASTn-based analyses for the three partial gene sequences supported taxonomic identity obtained from Bayesian phylogenetic analysis using concatenated data from nine representatives *P. musae* isolates (Supplementary Figure S1).

### Genetic Diversity

Genotyping revealed a total of 149 haplotypes among the ten populations analyzed. SSR profiles for all isolates are provided in Supplementary Table S1. An average genetic diversity of 4.06 was observed across the entire sample set (Table 1). Haplotype frequency varied across the samples, from eight haplotypes per population in Pops 7, 9, and 10 to 31 distinct haplotypes in Pop 3. Only three haplotypes were abundant and widely shared, between Pop 6 and Pop 7. When data was scaled based on the smallest population size using rarefaction curves (*n* = 8), the lowest allelic richness was found in Pop 7, with only 5.87 expected haplotypes. Lowest genotypic diversity was also observed in this population, with a Shannon Wiener index value of 1.95. Pops 4, 6, 8, 9, and 10 presented intermediate diversity values, with Pops 1, 2, 3, and 5 showing the highest diversity for the Shannon–Weiner and Hill indexes, with evenness also closest to 1. According to AMOVA-based analysis, greatest genetic differentiation occurred among individuals within populations, accounting for 86% of the total variation (Table 2).

**TABLE 1:** Genetic diversity, richness, and evenness in *Pseudocercospora musae* populations.

Region	Population^a^	N^b^	Hap_obs_^c^	H_exp(8)_^d^	H’^e^	(LL–UL)^f^	N_1_^g^	(LL-UL)^f^	E_5_^h^
1	Pop 1	21	21	8.00	3.04	(2.79–3.30)	21.00	(18.16–23.84)	1.000
	Pop 2	21	20	7.86	2.98	(2.73–3.23)	19.66	(16.62–22.70)	0.974
	Pop 3	31	31	8.00	3.11	(2.89–3.33)	31.00	(27.45–34.55)	1.000
	Pop 4	10	10	8.00	2.30	(1.97–2.63)	10.00	(7.98–12.02)	1.000
	Pop 5	18	18	8.00	2.89	(2.64–3.14)	18.00	(15.22–20.78)	1.000
2	Pop 6	22	16	7.04	2.65	(2.36–2.94)	14.15	(11.41–16.89)	0.844
	Pop 7	13	8	5.87	1.95	(1.58–2.32)	7.04	(5.20–8.88)	0.871
	Pop 8	9	9	8.00	2.20	(1.85–2.55)	9.00	(7.12–10.88)	1.000
3	Pop 9	9	8	7.22	2.04	(1.63–2.45)	7.70	(5.80–9.60)	0.948
4	Pop 10	8	8	8.00	2.08	(1.69–2.47)	8.00	(6.16–9.84)	1.000
Total	10	162	149		4.06				0.928

*^*a*^Populations analyzed: Distrito Federal – Pop 1: Estação Biológica (EB); Pop 2: Embrapa Cerrados (CPAC); Pop 3: Fazenda Canaã (FC); Pop 4: São João da Aliança (SJA); Pop 5: Chácara Santos (STO). Bahia; Pop 6: Bom Jesus da Lapa (BJL); Pop 7: Tancredo Neves (TaNe); Pop 8: Itajuípe (ITJ). Minas Gerais; Pop 9: Lavras (LV). Rio Grande do Norte; Pop 10: Baraúna (BRN). ^*b*^Number of individuals sampled; ^*c*^Number of observed haplotypes; ^*d*^Number of expected genotypes based on the rarefaction curve for a sample of eight individuals; ^*e*^Shannon–Wiener Index; ^*f*^Numbers in parentheses indicate the confidence interval calculated from bootstrapping with 1000 replicates; ^*g*^Hill’s index; ^*h*^E_5_ evenness index calculated by (G - 1)/(N_1_ - 1).*

**TABLE 2:** Analysis of Molecular Variance (AMOVA) for *Pseudocercospora musae* populations based on 15 simple-sequence repeat loci.

Source of variation	d.f.	Variance components (σ)	Percentage of variation	Φ-statistic
Between regions	3	0.32	8.059	0.13
Between populations	6	0.23	5.801	0.06
Within populations	152	3.48	86.13	0.08
Total	161	4.04	100.00	

*d.f., degrees of freedom.*

### Population Differentiation

Analysis of the *D*_*EST*_ index between population pairs (Table 3) generally revealed low genetic differentiation between geographically close populations, such as in the Distrito Federal (e.g., *D*_*EST*_ = 0.04 between EB and CPAC, 22 km distance), and high genetic differentiation between geographically distant populations (e.g., *D*_*EST*_ = 0.14 between EB and SeLa-BA, 1220 km distance). A lack of differentiation, however, was also apparent among certain populations, despite their considerable geographical distance. For example, for the two most geographically separate populations (Pop 10, Rio Grande do Norte and Pop 9, Minas Gerais), which are approximately 1900 km apart, a value of zero was observed for the D statistic.

**TABLE 3:** Genetic differentiation of *Pseudocercospora musae* populations estimated by the *D*_*EST*_ index (inferior triangle) and gene flow (*N*_*m*_) (superior triangle).

Populations^a^	Pop 1	Pop 2	Pop 3	Pop 4	Pop 5	Pop 6	Pop 7	Pop 8	Pop 9	Pop 10
Pop 1	–	13.62	499.50	499.50	499.50	4.24	3.13	6.44	499.50	6.49
Pop 2	0.04	–	499.50	7.97	73.03	5.60	3.25	84.25	499.50	5.23
Pop 3	0.00	0.00	–	499.50	499.50	4.41	3.73	9.13	499.50	8.95
Pop 4	0.00	0.06	0.00	–	499.50	4.26	3.57	14.84	499.50	11.61
Pop 5	0.00	0.01	0.00	0.00	–	3.62	4.15	8.97	499.50	8.92
Pop 6	0.11	0.08	0.10	0.11	0.12	–	61.23	499.50	499.50	5.86
Pop 7	0.14	0.13	0.12	0.12	0.11	0.01	–	499.50	499.50	10.71
Pop 8	0.07	0.01	0.05	0.03	0.05	0.00	0.00	–	499.50	499.50
Pop 9	0.00	0.00	0.00	0.00	0.00	0.00	0.00	0.00	–	499.50
Pop 10	0.07	0.09	0.05	0.04	0.05	0.08	0.04	0.00	0.00	–

*^*a*^Populations analyzed: Distrito Federal – Pop 1: Estação Biológica (EB); Pop 2: Embrapa Cerrados (CPAC); Pop 3: Fazenda Canaã (FC); Pop 4: São João da Aliança (SJA); Pop 5: Chácara Santos (STO). Bahia; Pop 6: Bom Jesus da Lapa (BJL); Pop 7: Tancredo Neves (TaNe); Pop 8: Itajuípe (ITJ). Minas Gerais; Pop 9: Lavras (LV). Rio Grande do Norte; Pop 10: Baraúna (BRN).*

### DAPC Cluster Analysis

**FIGURE 2:**
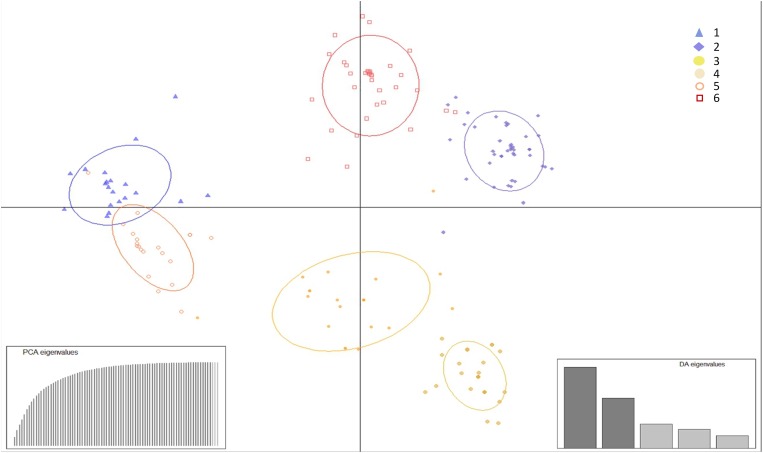
Discriminant analysis of principal components (DAPC) of *Pseudocercospora musae* isolates from different locations based on SSR loci. Each colored dot in the scatter plot represents an individual, with *k* the number of clusters (assuming *k* = 6). Isolates are clustered according to the probability of inclusion in the group. PCA and DA eigenvalues are illustrated in the enclosed barplots.

**FIGURE 3:**
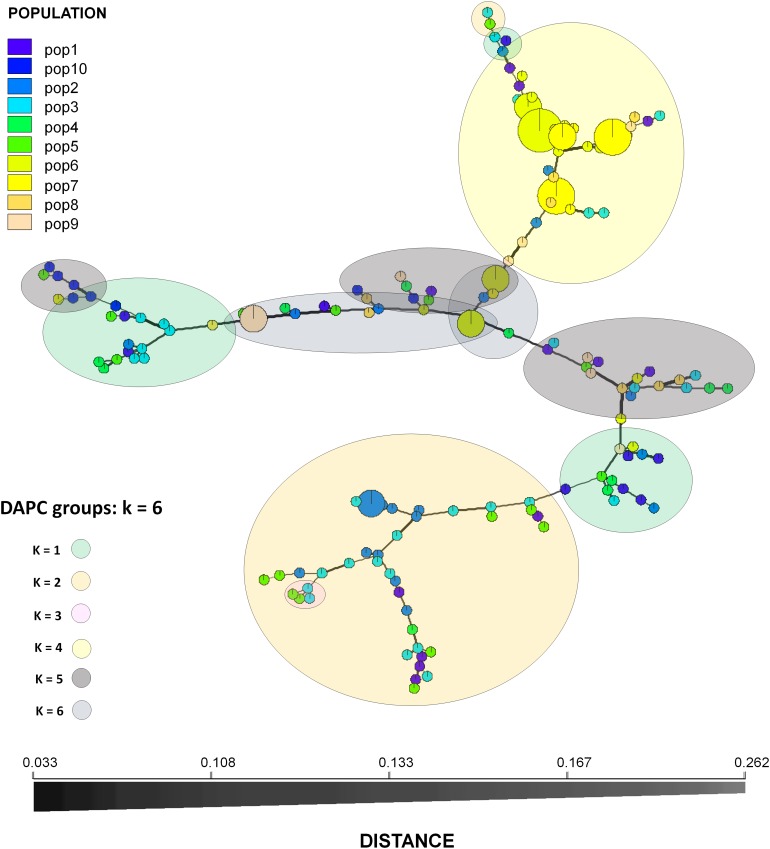
Minimum spanning network of the populations (162 isolates). Circles are separated by names of Multilocus genotypes (MLGs). Each circle represents a unique MLG and the colors the sampling sites. Circle size is proportional to MLG frequency. Edge widths and shading represent relatedness of MLGs based on Bruvo’s genetic distance. Edge length is arbitrary.

Population structure determination through DAPC cluster analysis revealed six separate groups (*k* = 6) (Figure 2). Clusters 1, 2, and 3 were represented by isolates from the vicinity of Brasilia, DF, cluster 4 by isolates from Bahia, and clusters 5 and 6 by isolates from Brasilia, Minas Gerais, Bahia and Rio Grande do Norte. The majority of isolates from cluster 6 originated from CPAC, with only one or two isolates originating from each of the populations FC, SJA, STO, BA, and RN (Figure 3).

### Sexual Recombination and Linkage Disequilibrium

Primers were efficient for amplification of mating type idiomorphs from *P. musae*, with a single amplicon produced for each isolate (400 bp for MAT1-1-1 and 700 bp for MAT1-2-1). Sequences for 155 of the isolates (Supplementary Table S1) showed 100% identity to the two *P. musae* mating type idiomorphs based upon BLASTn analyses against sequences in GenBank. Analysis of frequencies using the chi-square (χ2) test revealed that, in general, the studied populations did not show significant deviation from the expected 1:1 frequency of MAT1-1-1 and MAT1-2-1 idiomorphs, consistent with regular cycles of sexual reproduction in these populations (Table 4). In the case of Pop 5 from Chácara Santos (STO) in the Distrito Federal, however, the frequency for MAT1-1-1 observed was significantly higher than that of the idiomorph MAT1-2-1. Multilocus linkage-disequilibrium, using the indices of association estimated for each population *r*^–^*_*d*_* and *I*_*A*_, based on SSR loci was also employed to determine the contribution of sexual reproduction or clonal spread in the populations, with index values presented in Table 4. Evidence to reject the null hypothesis of linkage equilibrium under random mating, or sexual recombination, was observed in populations 3, 6, 7, and 9, with values above zero significant at the 1% probability level.

**TABLE 4:** Mating-type segregation in *Pseudocercospora musae* isolates from each population.

Region	POP	MAT1-1:MAT1-2 Frequency	χ^2^	*I*_*A*_	*r*^–^*_*d*_*
1	Pop 1	33.33:66.66	2.3330^ns^	−0.066^ns^	−0.006^ns^
	Pop 2	57.10:42.80	0.4286^ns^	0.306*	0.026*
	Pop 3	53.80:46.16	0.1538^ns^	0.176*	0.014**
	Pop 4	30.00:70:00	1.6000^ns^	0.126^ns^	0.011^ns^
	Pop 5	77.77:22.22	5.5556**	0.016^ns^	0.001^ns^
2	Pop 6	50.09:40.90	0.7273^ns^	0.782**	0.073**
	Pop 7	45.45:54.54	0.0909^ns^	1.297**	0.131**
	Pop 8	66.66:33.33	1.0000^ns^	0.259^ns^	0.033^ns^
3	Pop 9	33.33:66.66	1.0000^ns^	1.709**	0.143**
4	Pop 10	62.50:37.50	0.5000^ns^	0.421^ns^	0.050^ns^
Total	10	52.90:47.09	0.5226^ns^	0.166	0.012

^*ns*^not significant; *Significant at 5% probability level; **Significant at 1% probability level; *I*_*A*_ and *r*^–^*_*d*_* – Linkage disequilibrium calculated by 1000 permutations.

### DMI Fungicide Sensitivity and *CYP51* Nucleotide Sequence Variability

Sensitivity of *P. musae* to DMI fungicides cyproconazole, epoxiconazole, fluquinconazole, pyrifenox, prochloraz, propiconazole, tebuconazole, and triadimenol was analyzed along with the presence of CYP51 target site alterations in selected *P. musae* isolates. These were sampled from three populations in the Distrito Federal (Pops 2, 4, and 5; Figure 1). Analyses revealed five isolates from population 4 (SJA) showing generally higher EC_50_ values for all the different DMI fungicides tested in comparison to the isolates from populations 2 (CPAC) and 5 (STO) (Table 5).

**TABLE 5:** Demethylation inhibitor fungicide sensitivity profiles (EC_50_ values in μg ml^–1^) in *Pseudocercospora musae* isolates.

Isolate^1^	Epoxiconazole^2^	Tebuconazole	Triadimenol	Cyproconazole	Propiconazole	Fluquinconazole	Prochloraz	Pyrifenox
7CPAC	0.0044 ± 0.0007	0.021 ± 0.000	0.270 ± 0.008	0.042 ± 0.009	0.0049 ± 0.0000	0.0048 ± 0.0002	0.0022 ± 0.0003	0.0051 ± 0.0001
8CPAC	0.0050 ± 0.0006	0.028 ± 0.031	0.324 ± 0.018	0.048 ± 0.004	0.0059 ± 0.0002	0.0051 ± 0.0001	0.0020 ± 0.0001	0.0051 ± 0.0015
12CPAC	0.0049 ± 0.0012	0.030 ± 0.001	0.324 ± 0.002	0.055 ± 0.005	0.0063 ± 0.0003	0.0050 ± 0.0009	0.0035 ± 0.0010	0.0081 ± 0.0003
2SJA	0.0141 ± 0.0010	0.160 ± 0.066	0.740 ± 0.059	0.111 ± 0.002	0.0264 ± 0.0004	0.0124 ± 0.0003	0.0098 ± 0.0011	0.0479 ± 0.0112
4SJA	0.0079 ± 0.0016	0.119 ± 0.003	0.666 ± 0.002	0.081 ± 0.023	0.0125 ± 0.0006	0.0108 ± 0.0001	0.0045 ± 0.0017	0.0363 ± 0.0046
6SJA	0.0136 ± 0.0050	0.186 ± 0.042	0.915 ± 0.316	0.134 ± 0.037	0.0208 ± 0.0084	0.0124 ± 0.0003	0.0109 ± 0.0005	0.0600 ± 0.0162
9SJA	0.0050 ± 0.0006	0.019 ± 0.008	0.219 ± 0.013	0.052 ± 0.007	0.0065 ± 0.0015	0.0048 ± 0.0001	0.0037 ± 0.0015	0.0099 ± 0.0031
12SJA*	0.0127	0.329	1.77	0.295	0.0299	0.0188	0.0111	0.0795
13SJA	0.0112 ± 0.0030	0.272 ± 0.011	1.39 ± 0.045	0.144 ± 0.052	0.0288 ± 0.0038	0.0206 ± 0.0020	0.0125 ± 0.0005	0.0618 ± 0.0047
12STO	0.0052 ± 0.0003	0.022 ± 0.003	0.231 ± 0.019	0.038 ± 0.001	0.0046 ± 0.0001	0.0058 ± 0.0013	0.0026 ± 0.0003	0.0089 ± 0.0000
18STO	0.0079 ± 0.0011	0.025 ± 0.000	0.230 ± 0.015	0.039 ± 0.013	0.0063 ± 0.0005	0.0057 ± 0.0010	0.0025 ± 0.0003	0.0081 ± 0.0017
22STO	0.0054 ± 0.0004	0.020 ± 0.002	0.284 ± 0.026	0.046 ± 0.000	0.0049 ± 0.0000	0.0044 ± 0.0001	0.0037 ± 0.0004	0.0072 ± 0.0020

^1^Evaluated strains originated from populations: CPAC (pop 2); SJA (pop 4); STO (pop 5); ^2^Average EC_50_ data with standard deviation, based on measurements across multiple fungicide dilution series for each isolate-fungicide combination. *Data based on measurements on only a single fungicide dilution series.

**FIGURE 4:**
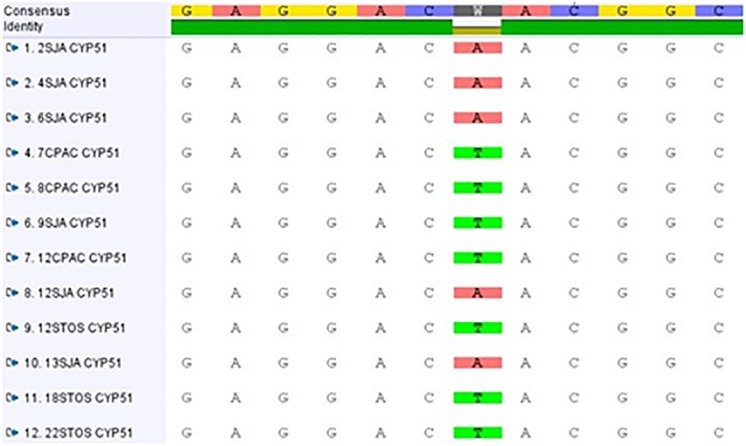
Alignment of *CYP51* gene sequences of evaluated *Pseudocercospora musae* isolates from three locations [Pop 2: Embrapa Cerrados (CPAC); Pop 4: São João da Aliança (SJA); Pop 5: Chácara Santos (STO)].

Using primer pair MMFOR2/REV1, a fragment of 1702 bp was amplified and sequenced in all isolates (see nucleotide positions 550-2251 in GenBank accessions MF521833 and MF521834). Given the high nucleotide sequence similarity (89%) to the *P. fijiensis* CYP51B gene coding sequence (GenBank XM_007930561), it was evident that this fragment covered CYP51 codon positions 7 to 507, after removal of primer sequences. This region of the protein shows a high level of homology with *P. fijiensis* CYP51B (96.2%) and spans all positions that have been reported to affect azole binding (Mair et al., 2016). When sequences of the 12 isolates were compared, two nucleotide changes were observed. One mutation observed in isolate CPAC7 was synonymous, with a codon change of TTG into CTG (leucine) at position 154. The other mutation, a T to A change, was a non-synonymous substitution resulting in the replacement of tyrosine (Y (TAC)) by asparagine (N (AAC)) at codon 461 (Y461N). Y461N was present in all five azole insensitive isolates from Pop 4, but not detected in the seven azole sensitive isolates from Pops 2, 4, and 5 (Figure 4). Based on the EC_50_ values, the resistance factors (average EC_50_ value of insensitive isolates divided by average EC_50_ value of sensitive isolates) associated with CYP51 Y461N were 2.2, 3.0, 3.3, 3.4, 4.1, 4.2, 7.6, and 9.1 for epoxiconazole, fluquinconazole, cyproconazole, prochloraz, triadimenol, propiconazole, pyrifenox and tebuconazole, respectively. Using primer pairs MMFOR1/REV5 and MMFOR8/REV8, which amplified fragments of 671 and 187 bp, respectively, the CYP51 gene was further sequenced in two DMI fungicide-sensitive isolates (8CPAC and 9SJA) and two insensitive isolates (2SJA and 18STO). Assembly and alignment of sequences from 539 bp upstream of the start codon to the last codon revealed no further nucleotide alterations in either the CYP51 promoter region or the coding sequence among the comparative isolates.

## Discussion

Sigatoka leaf spot has been present in Brazil for almost 70 years, causing significant impact on banana production across the country (Matos and Cordeiro, 2011). An increased understanding of the population genetics of the pathogen is required for the development of appropriate disease management strategies across growing regions.

In the current study, data analysis revealed significant differences in genetic diversity among both populations from the same state and those from different states, with Shannon Wiener index values ranging from 1.95 to 3.11. Differences between populations in terms of diversity, richness and evenness may be due to population arrangement and age, as well as population size. Sample size has previously been identified as a factor influencing genetic differentiation between African and Latin American-Caribbean *P. musae* populations (Hayden et al., 2003). In previous analysis of the diversity of *P. musae* populations in Australia, variation of Nei’s genetic diversity (*H*) also ranged from 0.142 to 0.360, from smallest to largest populations, respectively (Hayden et al., 2005). Considerable diversity has been reported in populations of other Dothideomycete fungal pathogens, such as the black leaf streak pathogen *P. fijiensis* from Columbia (0.46) (Perea et al., 2005), as well as in *Z. tritici* populations from Saskatchewan (0.44) (Razavi and Hughes, 2004) and the United States (0.54) (Gurung et al., 2011). Recent investigation of the genetic structure of *P. musae* populations from Minas Gerais in Brazil also revealed high genetic diversity across the sampled area (0.71) (Gomes et al., 2017).

According to AMOVA results, the largest contribution to population diversity occurred within populations (86%), rather than among populations (5.8%). The lack of population structure and low differentiation of populations, as also observed by Peixouto et al. (2015), may be explained by the low genetic differentiation between population pairs, demonstrated by the frequently low *D*_*EST*_ values and high values of migrants per generation, distributing new alleles and contributing to the homogenization of certain populations. This is in agreement with what is expected for the disease, with dissemination able to occur locally (by rain splash) and over long distances (by air) (Hayden et al., 2003). In previous studies in *P. musae*, *F*_*ST*_ metric values from 0.04 to 0.45 were reported in populations from Australia (Hayden et al., 2005). Similarly, for *P. fijiensis, F*_*ST*_ values in populations from Colombia ranged from 0.07 to 0.26 (Perea et al., 2005). *F*_*ST*_ values from 0.03 to 0.58 were also observed in populations of *P. fijiensis* from Latin American countries (Rivas et al., 2004), with the higher values reflecting sample origin from different countries. In our study, given the large physical distances (470–1,900 km) among the *P. musae* isolates from the analyzed states, a high diversity among all the populations was perhaps expected, as supported by DAPC analysis. Diversity within populations may also be explained by the presence of sexual recombination occurring at a local level, which was confirmed by the mating-type ratio of 1:1 observed for most populations, together with recombination signals for different populations which were identified based on linkage disequilibrium analysis.

The number of unique haplotypes in populations, together with corresponding genetic diversity and observed equal frequency of mating type, provide evidence for frequent sexual recombination in this species. Whilst such 1:1 frequencies of mating type gene idiomorphs have also been reported for populations of *P. musae* from Minas Gerais in Brazil (Gomes et al., 2017), as well as for *P. fijiensis* isolates from different states in Brazil (Queiroz et al., 2013), from Mexico (Conde-Ferráez et al., 2010) and for *Z. tritici* isolates from different continents (Zhan et al., 2002), differences in frequency can also occur in specific growing regions, potentially associated with aggressiveness (Manzo-Sánchez et al., 2019). Sexual reproduction is important in heterothallic fungal species, contributing to gene flow among individuals, generating new allelic combinations within populations and the ability to adapt to unfavorable conditions. For both *P. fijiensis* and *P. musae*, gene flow within banana fields occurs by rain splash of asexual conidia produced on leaves, whilst movement between banana fields will be determined by dispersal of airborne sexually produced ascospores ejected from pseudothecia (Hayden et al., 2003, 2005). Although ascospore spread in *P. musae* reduces genetic differentiation between geographically distant populations, any migration of viable spores over distances greater than 50 km is likely to be limited (Hayden et al., 2005), as ascospores are unlikely to survive long periods of UV irradiation (Parnell et al., 1998). Short distance dispersal by ascospores, however, may explain the low differentiation among the tested populations of the Distrito Federal, which were all sampled within a distance of 50 km. Interestingly, low genetic differentiation was also reported among populations of *P. musae* from large banana production areas in Australia, with the distance between populations greater, in the region of 1000 km (Hayden et al., 2005). If not due to ascospore spread, such reduced diversity among geographically distant populations may be due to transportation of infected germplasm material among *Musa* growing regions (Brown and Hovmoller, 2002; Rivas et al., 2004).

Although sexual reproduction was an evident process in the *P. musae* populations analyzed, analysis of multilocus linkage disequilibrium in some populations suggests that asexual reproduction may also play an important role in the genetic structure of these populations, supporting documented evidence of conidial dispersal within banana blocks (Hayden et al., 2005). The final result of a combination of both reproduction processes is potentially advantageous for the pathogen. While sexual reproduction creates new genotypes through recombination, frequent asexual reproduction cycles can disseminate a new pathogen variant isolate that has arisen by selective pressure. In the case of fungicide resistance development in populations, the conventionally adopted site-specific DMI fungicides for pathogen control are more likely to be overcome by such combined mechanisms of genetic variability.

Selected *P. musae* isolates characterized for DMI sensitivity were collected from agroecological zones in the Distrito Federal where control of Sigatoka leaf spot is achieved with alternating applications of DMI and quinone outside inhibitor (QoI) fungicides (Gisi et al., 2002). Data revealed isolates from São João da Aliança with higher levels of insensitivity to all DMIs tested, originating from the two *M. acuminata* cultivars Prata and Cavendish Grande naine present in the location. As SSR analysis revealed different profiles, these isolates were not considered to be clonal (Supplementary Table S1). The presence of a *CYP51* mutation resulting in Y461N was confirmed in all the insensitive isolates. Although the point mutation CYP51 Y461N has not been reported for *P. fijiensis*, a different amino acid alteration at the same position (Y461D) has been linked with high levels of DMI insensitivity (Cañas-Gutiérrez et al., 2009). An identical alteration of CYP51, Y459N, has also been found in *Z. tritici*, albeit at low frequencies and in combination with other mutations (Cools and Fraaije, 2013). The codon position of 459 in *Z. tritici* is slightly different to position 461 in *P. musae* and *P. fijiensis* due to differences in the protein structure (Mair et al., 2016). Molecular modeling studies with *Z. tritici* CYP51 has shown that substitutions at positions 459–461 cause azole resistance by moving residues V136 (V135 in *P. musae*) and or Y137 (Y136 in *P. musae*), which are key residues for azole binding, further from the docked azoles (Mullins et al., 2012). Single CYP51 target alterations are usually linked to low levels of DMI insensitivity, although based on experience with other species, such as *Z. tritici* and *P. fijiensis*, it is likely that multiple mutations will evolve which, in combination with each other and with other resistance mechanisms, such as overexpression of CYP51 and efflux pumps, will result in higher levels of resistance to fungicides (Cools et al., 2012; Omrane et al., 2015). With QoI resistance already developed in *P. musae* in different geographic regions in Australia (Grice et al., 2013), it is therefore essential to monitor the DMI and QoI sensitivity profiles and CYP51 polymorphisms across further Brazilian *P. musae* field populations, to enable baseline information on resistance emergence and distribution across *Musa* growing regions.

An increased understanding of the genetic diversity of pathogen populations, together with monitoring of the evolution and spread of DMI and QoI resistance, is fundamental for the development of optimal integrated management strategies for Sigatoka leaf spot, based on host resistance breeding and rational design of fungicide resistance management strategies involving agrochemical mixtures or alternation regimes.

## Data Availability Statement

Representative sequence data employed in molecular identification of *P. musae* is available in GenBank under accession numbers: KP996493, KP996494, and KP996495.

## Author Contributions

RM, FB, and JS planned the experiments. FB and JS conducted the isolate sampling, molecular identification, and characterization of mating types and haplotypes. FB, VA, YP, SO, CF, FH, and EA participated in data analysis. FB and BF performed fungicide sensitivity bioassays. RM conceived the study, participated in characterization of mating types and haplotypes, and drafted the manuscript. All authors have contributed to, read, and approved the final manuscript.

## Conflict of Interest

The authors declare that the research was conducted in the absence of any commercial or financial relationships that could be construed as a potential conflict of interest.

## Footnotes

1 http://blast.ncbi.nlm.nih.gov/Blast.cgi

2 http://genome.jgi.doe.gov

## Supplementary Material

FIGURE S1 Unrooted Bayesian consensus tree of concatenated data from the ITS, ACT, and HIS gene sequences of nine representative *Pseudocercospora musae* isolates. The following models were employed: for ITS: K80 + I; for ACT: K80 + I; and for HIS: HKY + G. Posterior probability values are shown.

TABLE S1 *Pseudocercospora musae* isolate origin, SSR profile, and mating type.
