# Supplementary figures and images for: Genetic Diversity and Azole Fungicide Sensitivity in Pseudocercospora musae Field Populations in Brazil
Source: Front Microbiol. 2020 Feb 4;11:99. doi: 10.3389/fmicb.2020.00099 (PMC7011104; doi:10.3389/fmicb.2020.00099)

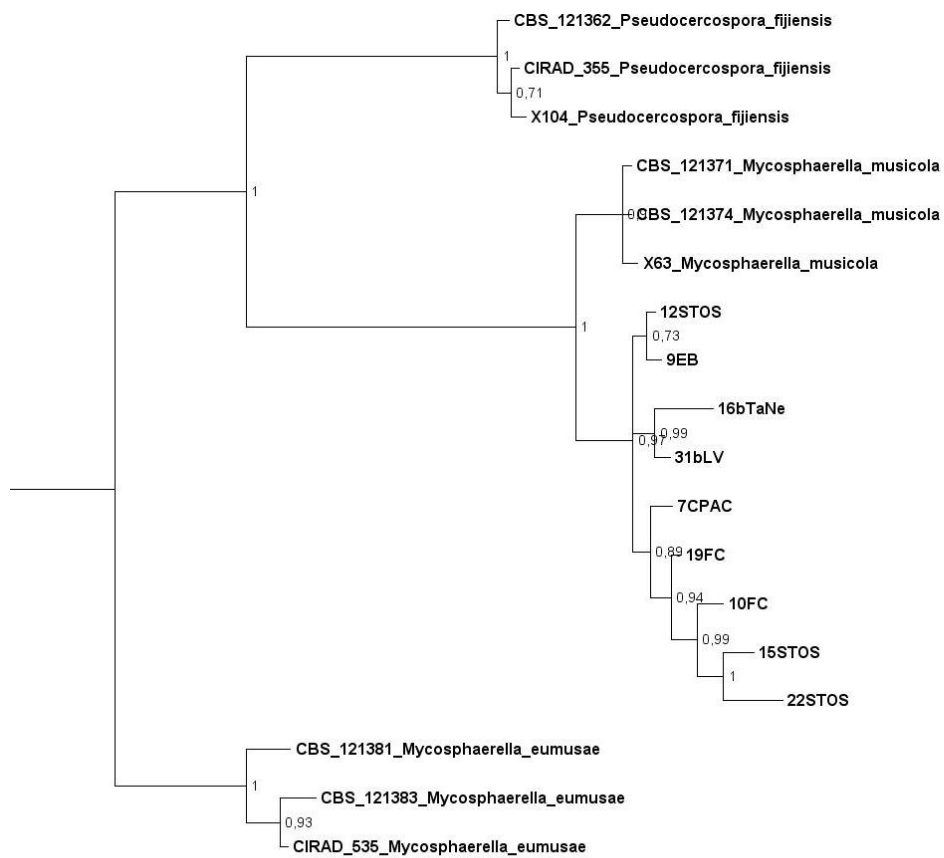

0.02

Supplement: FIGURE S1 — Unrooted Bayesian consensus tree of concatenated data from the ITS, ACT, and HIS gene sequences of nine representative Pseudocercospora musae isolates. The following models were employed: for ITS: K80 + I; for ACT: K80 + I; and for HIS: HKY + G. Posterior probability values are shown. [file Image_1.pdf]
